# Phonological processing in deaf signers and the impact of age of first language acquisition

**DOI:** 10.1016/j.neuroimage.2007.12.047

**Published:** 2008-04-15

**Authors:** Mairéad MacSweeney, Dafydd Waters, Michael J. Brammer, Bencie Woll, Usha Goswami

**Affiliations:** a Behavioural and Brain Sciences Unit, UCL Institute of Child Health, 30 Guilford Street, London WC1N 1EH, UK; b Deafness, Cognition and Language Research Centre, Department of Human Communication Science, University College London, London WC1H 0PD, UK; c Institute of Psychiatry, King's College London, De Crespigny Park, London SE5 8AF, UK; d Centre for Neuroscience and Education, Faculty of Education, University of Cambridge, 184 Hills Road, Cambridge CB2 2PQ, UK

**Keywords:** Deaf, Sign language, Plasticity, Phonology, Phonological awareness, Age of acquisition, Inferior frontal gyrus, Rhyming, Pictures

## Abstract

Just as words can rhyme, the signs of a signed language can share structural properties, such as *location*. Linguistic description at this level is termed phonology. We report that a left-lateralised fronto-parietal network is engaged during phonological similarity judgements made in both English (rhyme) and British Sign Language (BSL; location). Since these languages operate in different modalities, these data suggest that the neural network supporting phonological processing is, to some extent, *supramodal.* Activation within this network was however modulated by language (BSL/English), hearing status (deaf/hearing), and age of BSL acquisition (native/non-native). The influence of language and hearing status suggests an important role for the posterior portion of the left inferior frontal gyrus in speech-based phonological processing in deaf people. This, we suggest, is due to increased reliance on the articulatory component of speech when the auditory component is absent. With regard to age of first language acquisition, non-native signers activated the left inferior frontal gyrus more than native signers during the BSL task, and also during the task performed in English, which both groups acquired late. This is the first neuroimaging demonstration that age of first language acquisition has implications not only for the neural systems supporting the first language, but also for networks supporting languages learned subsequently.

## Introduction

**Fig. 1:**
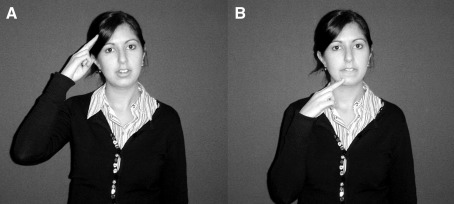
These BSL signs (A) NAME and (B) AFTERNOON differ only in location; handshape and movement are the same.

Phonology describes the level of analysis at which meaningless, contrastive units of language combine to form meaningful units. In spoken languages these are auditory/articulatory elements. Substitution of a single element creates a new lexical item, e.g., in English /pin/–/bin/. The same level of analysis has been applied to signed languages, where phonology is visual, with handshapes, movements and locations combined to form signs (Stokoe, 1960; Brentari, 1999; Sandler and Lillo-Martin, 2006). As with words, the substitution of just one element can create a new sign. For example, the BSL sign NAME is located at the forehead while AFTERNOON differs only in that it is located at the chin (see Fig. 1).

The primary aim of the current study was to examine whether the application of the term ‘phonology’ to signed languages has neurological as well as linguistic and psycholinguistic validity. We address, for the first time, whether similar neural processing is involved in phonological analysis of both signed and spoken languages. We asked participants to judge whether spoken word labels for pictures rhymed or not. Of the phonological parameters of signs, location is one of the primary factors determining whether signs are judged to be similar (Hildebrandt and Corina, 2002). Therefore, we also asked deaf participants to judge if BSL signs for pictured items shared the same location.

Studies of written rhyme judgement by hearing people report reliable activation of the posterior portion of the left inferior frontal gyrus (IFG) and the ventral premotor cortex (Broca's area; Sergent et al., 1992; Poldrack et al., 1999; Kareken et al., 2000; Lurito et al., 2000; Xu et al., 2001; Seghier et al., 2004; Burton et al., 2005). Temporary disruption of this region using transcranial magnetic stimulation (TMS) impairs phonological processing (Gough et al., 2005). Given the well-established role of the posterior IFG in speech production (Ojemann and Mateer, 1979), the contribution of the left posterior IFG and ventral premotor cortex to phonological processing is often attributed to articulatory processes or representations (Démonet et al., 1996). In addition to the left IFG, the left parietal lobe is also recruited during rhyme judgement tasks using written stimuli (e.g., Pugh et al., 1996; Lurito et al., 2000; Xu et al., 2001; Seghier et al., 2004). However, the precise role of this region in phonological processing remains unclear (see Eden et al., 2004) and will be addressed further in the Discussion section. If similar processing is required to make phonological similarity judgements about BSL and English, left inferior frontal and left parietal regions should be recruited during both tasks.

**Fig. 2:**
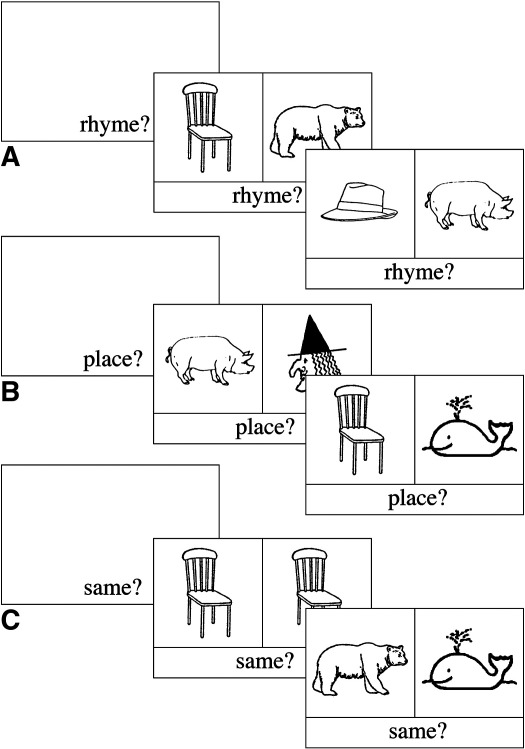
Schematic representation of order of events during the: (A) rhyme (English); (B) location (BSL); and (C) ‘same picture?’ judgement tasks. The first trial in each example block is a ‘yes’ trial and the second is a ‘no’ trial. In the actual task, trial order was randomised.

An important feature of the current study was the use of picture stimuli (see Fig. 2). This enabled the same stimuli and response requirements to be used in both the BSL and English tasks. This is not possible in studies contrasting comprehension (e.g., MacSweeney et al., 2002b) or production (e.g., Braun et al., 2001) of speech and sign. Picture stimuli also allowed us to tap individuals' own phonological representations of words and signs. Previous studies of rhyme judgement with hearing people have used auditory or written stimuli, which directly or indirectly provide the phonology of the item. To determine whether rhyme judgements in response to pictures elicit the pattern of activation reported in studies using written words, hearing participants were also tested. Including this group also allowed us to directly contrast activation patterns observed in deaf and hearing participants to determine the effect of hearing status on the neural systems supporting phonological processing.

A second aim of this study was to determine if the neural systems supporting language are influenced by the age of *first* language acquisition. To address this, deaf native and non-native signers were contrasted. Approximately 5% of deaf people are born to deaf, signing parents (Mitchell and Karchmer, 2004). Typically, these children learn a signed language as their native language, reaching acquisition milestones along the same timescale as hearing children acquiring speech (Morgan and Woll, 2002). However, for nearly all of the 95% of deaf children who are born to hearing parents (non-native signers), exposure to a signed language is delayed.

Deaf native and non-native signers should differ on *sign*-related tasks since this is the language acquired early or late. In addition, Mayberry et al. have shown that deaf native signers perform better than deaf non-native signers on grammaticality judgements of written *English* (Mayberry et al., 2002; Mayberry and Lock, 2003). In these studies, both groups encountered written English at the same age. However, what *did* differ between these groups was their early language experience. Native signers have a well-established first language which can facilitate the acquisition of a later learned language; non-native signers do not. For all deaf people, both native and non-native signers, exposure to spoken language is late and incomplete. This is because speechreading cannot provide full access to speech since many of the articulators of speech are invisible. From this perspective, Mayberry and Lock (2003) have argued that deaf non-native signers can be considered to have ‘no early language’. This is a very different situation to that of hearing people learning a signed language late since they already have a native spoken language. Studies contrasting early and late acquisition of signed language in hearing participants (cf., Newman et al., 2002) cannot therefore be generalised to the deaf population. We examined how the incomplete acquisition of a language early in life is reflected in the brain. Consistent with the behavioural findings of Mayberry et al., we predicted differences between deaf native and non-native signers, on both the BSL *and* English tasks, despite these groups' similar experience of English.

In summary, in the current study profoundly deaf and hearing adults made phonological similarity judgements in response to picture pairs (see Fig. 2). Participants judged if the spoken English labels *rhymed* or whether the BSL labels shared the same *location* (deaf group only). Activation during these experimental conditions was contrasted with a ‘same picture?’ control task. The following research questions were addressed: are the same neural networks recruited during phonological decisions based on signed and spoken language? What is the impact of age of signed language acquisition and hearing status on this network?

## Materials and methods

### Participants

Twenty-three deaf adults and 24 hearing adults were scanned. All were right-handed and had normal or corrected to normal vision. Of the deaf participants, one was excluded because of excessive movement in the scanner and another two were excluded because they did not complete both the sign and speech tasks. Therefore, 20 deaf and 24 hearing participants, matched on age and non-verbal IQ (Block Design, WAIS-R: *p* > 0.1), were included in the analyses (see Table 1 for participant characteristics). The deaf participants included in the fMRI study were selected from a larger sample of volunteers. To enhance the likelihood that they would perform the phonological judgement tasks well, those selected were good readers and had performed well on a test of rhyme ability in a previous test session outside the scanner. Therefore, the deaf participants included in the fMRI study were good readers (mean reading age = 15years, 6months) in comparison to the population mean for deaf people, generally considered to be approximately 9 or 10years (see Conrad, 1979; Allen, 1986; Holt, 1993). The deaf participants were also skilled speechreaders, outperforming the hearing participants on the Test of Adult Speechreading (Mohammed et al., 2003; *t* = 5.1, (34), *p* < 0.0005). Nevertheless, the hearing group were significantly better readers (Vernon-Warden, 1996: *t* = − 3.9, (42), *p* < 0.0005) and had a higher English vocabulary score (shortened version of the Boston Naming Test, Kaplan et al., 1983: *t* = − 4.1, (42), *p* < 0.0005) than deaf participants. These differences are accounted for in the fMRI data analyses.

All deaf participants reported being born profoundly deaf and audiograms obtained at the time of testing confirmed that all had a mean hearing loss greater than 92dB in the better ear over four octaves, spanning 500–4000Hz. All deaf participants encountered written English upon entering primary school, aged 4/5. Twelve of the deaf participants were native signers, having acquired BSL from their deaf, signing parents. The remaining eight deaf participants (non-native signers) had hearing parents. One native signer and one non-native signer reported attending schools which used a total communication approach, in which signs are used to support spoken English. The remaining 18 of the 20 deaf participants had attended ‘oral’ schools in which spoken English was the main form of communication. This educational approach was the norm for this generation of deaf adults in the UK, even for those who used BSL as their native language. Of the eight non-native signers, five learned BSL after leaving secondary school, aged 17 to 21. One participant learned BSL at their total communication primary school aged 4/5. Two other participants who attended oral schools reported learning BSL at school; one aged 4/5, the other aged 11. These participants will have been exposed to BSL by their deaf native signing classmates.

The deaf subgroups were well matched. There were no significant differences between deaf native and deaf non-native signers in age (*p* = 0.06), non-verbal IQ (*p* > 0.1), reading age (*p* > 0.1), English vocabulary (*p* > 0.1) or speechreading skill (*p* > 0.1). All participants gave informed, written consent to participate in the study, which was approved by the Institute of Psychiatry/South London and Maudsley NHS Trust Research Ethics Committee.

### Stimuli

The same pictures were presented in both the rhyme and location judgement tasks (*n* = 60). All pictures represented highly familiar/frequent, monosyllabic English words. Fifty-eight of the pictures were black and white line drawings, taken predominantly from the Snodgrass and Vanderwart (1980) standardised picture set and other language assessments. Two colour pictures were also included, to represent ‘red’ and ‘blue’.

#### Rhyme task (shared English phonology pairs)

Thirty pictures were combined as 15 rhyming pairs. Orthography was inconsistent so that rhyme decisions could not be based on spelling, (e.g., chair–bear, tail–whale; see Fig. 2A).

#### Location task (shared BSL phonology pairs)

The remaining thirty pictures were combined as 15 pairs sharing the same location when signed in BSL, but differing in handshape and movement (e.g., pig–witch; hat–cow, see Fig. 2B). Only signs touching the body or occurring in close proximity to it were considered to have a defined location. Participants were told that any pairs articulated in neutral space in front of the signer's body (see Stokoe, 1960) should receive a ‘no’ response.

The two sets of experimental pictures (rhyme and location pairs) were matched on familiarity (Coltheart, 1981) (*t* = 0.64, 53, *p* > 0.1) and concreteness (Coltheart, 1981) (*t* = − 1.1, 53, *p* > 0.1) and the English labels were matched on frequency (Kucera and Francis, 1967) (*t* = 0.92, 56, *p* > 0.1) and length (*t* = 1.5, 58, *p* > 0.1). The ‘no’ (non-shared phonology) trials were established by re-pairing the pictures from the complementary task. Rhyming pictures were re-paired such that there was no overlap in signed or spoken phonology to form the ‘no’ trials in the location task (e.g., chair–whale). Likewise, location pairs were re-paired to form the ‘no’ trials in the rhyme task (e.g., hat–pig). Thus, the same stimuli were presented in both tasks.

The stimuli used in the experimental conditions doubled as their own controls in the ‘same picture?’ control task. Fifteen of the pictures were presented as identical pairs (e.g., chair–chair, see Fig. 2C). Another 30 pictures were re-paired to form *different* picture trials. The labels for these items did not share any phonological features in either English or BSL. Thus, of the 60 pictures seen in both experimental conditions, 45 were also presented in the ‘same picture?’ control condition. Whether an item was first seen in the experimental or control condition was counterbalanced such that any repetition effects were balanced across conditions.

All participants performed a picture naming pre-test before the scan session. If an unexpected label was generated, the desired English word or BSL sign (deaf participants only) was supplied. Correct naming of these items was checked again at the end of the pre-test session.

### Design

Deaf participants performed the rhyme and location similarity judgement tasks in separate, counterbalanced runs. Hearing participants performed only the rhyme task. Each run consisted of six 30-s blocks of the experimental task (rhyme or location), alternating with six 30-s blocks of the ‘same picture?’ control task. Each run lasted 6 min.

In the English phonology task, participants were required to decide whether the English labels for two pictures rhymed. Deaf participants had already been involved in a behavioural study of rhyme awareness as part of a wider project. They were reminded of the concept of rhyme and were given examples and practice trials prior to the start of the experiment in the scanner. In the sign phonology task, signing participants were required to decide if the BSL labels for the two pictures shared the same location. The control condition was interleaved between the *same phonology*? task blocks. This consisted of deciding if two pictures were the same. The trials in each condition were half ‘yes’ trials and half ‘no’ trials. Subjects indicated their response using a two-choice button box.

A one-syllable task prompt appeared at the bottom of the screen, without a pair of pictures, for 2000ms at the beginning of each block (‘Rhyme?’—rhyme task; ‘Place?’—location task; ‘Same?’—picture matching task). The prompt remained on the screen throughout the block. Each pair of pictures was presented for 5s. This relatively long presentation duration was selected on the basis of pilot studies in which deaf people made self-paced rhyme decisions in response to pictures. The inter-stimulus interval was 500ms. Each 30-s block was a mixture of five ‘yes’ and ‘no’ trials (see Fig. 2).

### fMRI parameters

Gradient echo echoplanar MRI data were acquired using a 1.5-T GE NVi MR system (General Electric, Milwaukee, WI, USA) using a standard quadrature head coil. Head movement was minimised by positioning the participant's head between cushioned supports. One hundred and twenty T_2_^⁎^-weighted images depicting BOLD contrast were acquired during one experimental session at each of 38 near-axial 3mm thick planes parallel to the intercommissural (AC–PC) line: 0.3mm interslice gap; TR = 3s, TE = 40ms; flip angle = 90°). The field of view for the fMRI runs was 240mm, and the matrix size was 64 × 64, with a resultant in-plane voxel size of 3.75mm. An inversion recovery EPI dataset was also acquired to facilitate registration of individual fMRI datasets to Talairach space (Talairach and Tournoux, 1988). This comprised 43 near-axial 3mm slices (0.3mm interslice gap), which were acquired parallel to the AC–PC line (TR = 16s; TE = 80ms; TI = 180ms; flip angle = 90°). The field of view for the EPI dataset was 240mm, and the matrix size was 128 × 128, with a resultant in-plane voxel size of 1.875mm.

### fMRI data analysis

The fMRI data were analysed using an in-house non-parametric software package (XBAM_v3.2) which uses standard preprocessing steps: realignment, normalisation, baseline correction, spatial smoothing, and GLM parameter estimation using a combination of gamma variate basis functions (for details see Brammer et al., 1997; Bullmore et al., 1999, 2001; Suckling and Bullmore, 2004). The data were realigned to minimise artefacts due to subject motion. First, a template was computed by averaging the image intensity over all time points at each voxel. The 3D volumes at each time point for each participant were then realigned to the template by computing the rigid body motion parameters (3 rotations, 3 translations) that maximised the correlation between each volume and the template. Normalisation was conducted using an affine transform and by computing the parameter set that maximised the correlation between the template image (in standard space—Talairach and Tournoux) and the image to be normalised. The data were then smoothed using a Gaussian filter (FWHM 7.2mm). Experimental responses were then analysed by convolving the experimental design with two gamma variate functions (peak responses four and eight seconds) with delays chosen to span the likely range of BOLD delays and computing the least squares fit of the resulting convolution to the time series at each voxel. A goodness of fit statistic was derived by calculating the ratio between the sum of squares due to the model fit and the residual sum of squares (SSQ ratio). The value of this statistic was then tested for significance using the wavelet-based time series permutation method (Bullmore et al., 2001; Suckling and Bullmore, 2004).

#### Group analysis

Data were transformed into standard space (Talairach and Tournoux, 1988). Voxel size in standard space was 3.3 × 3.3 × 3.3mm. Significant activations were identified using data-driven significance testing of the median activations at each voxel over all members of the group (Brammer et al., 1997). Median statistics were used to minimise outlier effects in the group sizes normally used in fMRI studies. Analysis was extended to the cluster level with the clusterwise false positive threshold set to less than one across the whole brain (Bullmore et al., 1999). Since the XBAM analysis method takes into account first level as well as second level variance, it resembles what Thirion et al. (2007) have called a “pseudo mixed effects analysis”.

#### Group differences

Differences in activation between groups and conditions were assessed by fitting the following linear model to the data at each voxel, **Y** = *a* + *b***X** + ***e***, where ***Y*** is the vector of BOLD effect sizes for each individual, **X** is the contrast matrix for the particular inter-condition/group contrasts required, *a* is the mean effect across all individuals in the various conditions/groups, *b* is the computed group/condition difference, and ***e*** is a vector of residual errors. The model was fitted by minimising the sum of absolute deviations to reduce outlier effects. The null distribution of *b* was computed by permuting data between conditions (assuming the null hypothesis of no effect of experimental condition) and refitting the above model. Group difference maps were computed as described above at voxel or cluster level by appropriate thresholding of the null distribution of *b*.

#### Conjunction analysis

Conjunction analyses were carried out to identify brain regions in which there was consistent activation across tasks. First, the minimum SSQ ratio (effect/error) at each voxel across conditions was determined. This measure was then tested (at appropriate voxelwise and clusterwise *p*-values), as described above under Group analysis, to determine whether it was significantly different from zero. Brain areas showing significant levels of activation were considered to show significant conjunctions of brain activation.

## Results

### Behavioural data

See Table 2 for accuracy and reaction time data.

#### Deaf participants only

A mixed-model ANOVA was conducted on the accuracy data (Task (rhyme/location/control) × Group (native/non-native signers)). A main effect of Task indicated that the control task was performed better than both experimental tasks (*F*(2,36) = 25.5, *p* < 0.0005). There was no significant effect of Group and no interaction. Excluding the control task from the ANOVA yielded no significant main effects and no interaction. Thus, deaf native and non-native signers were equally accurate on both the rhyme and location tasks.

The same mixed-model ANOVAs were applied to the reaction time data. Deaf participants were faster on the control than experimental tasks (*F*(2,36) = 211.7, *p* < 0.0005). There were no further significant effects. When the control task was omitted from the model, a main effect of Task (*F*(1,18) = 9.7, *p* < 0.01) indicated faster reaction times to the rhyme than location task.

#### All deaf versus all hearing participants performing the rhyme task

A mixed-model ANOVA was conducted on the accuracy data (Task (rhyme/control) × Group (deaf/hearing)). A main effect of Task indicated better performance in the control than rhyme task (*F*(1,42) = 52.5, *p* < 0.0005). A main effect of Group indicated better performance by hearing than deaf participants (*F*(1,42) = 13.4, *p* < 0.002). This was qualified by a significant interaction (*F*(1,42) = 11.4, *p* < 0.005) indicating that the hearing group performed better than the deaf group on the rhyme task, with no difference on the control task.

With regard to the reaction time data, a main effect of Task (*F*(1,42) = 744.7, *p* < 0.0005) indicated faster responses on the control than rhyme task. A significant interaction (*F*(1,42) = 11.5, *p* < 0.005) indicated slower performance by deaf than hearing participants to the rhyme task, but no group difference on the control task.

### fMRI data

#### Rhyme and location similarity judgements in deaf participants only

**Fig. 3:**
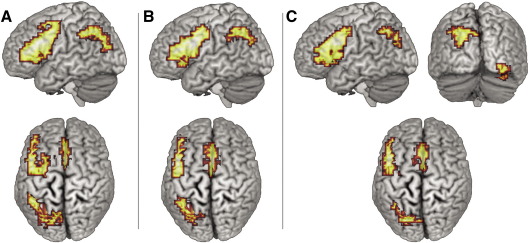
Activation, relative to the ‘same picture?’ control task, during the: (A) location task in deaf participants (*n* = 20); (B) rhyme task in deaf participants (*n* = 20); (C) rhyme task in hearing participants (*n* = 24). Voxelwise *p* < 0.025; clusterwise *p* < 0.01. Activated voxels up to 20mm beneath the cortical surface are displayed.

To identify neural systems involved in phonological processing of sign and speech in the deaf group, data from all deaf participants were combined. Analysis of the rhyme (English) and location (BSL) tasks separately, relative to the control task (voxelwise *p* = 0.025; clusterwise *p* = 0.01), resulted in remarkably similar patterns of activation (see Figs. 3A and B/Table 3).

A conjunction analysis was performed to clarify the overlap in activation between the two phonological tasks (English rhyme and BSL location) in deaf participants (voxelwise *p* = 0.05; clusterwise *p* = 0.025). As in the individual task analyses, a network consisting of three regions was identified. The most extensive activation was in the left frontal cortex (19.41cm^3^ volume; *X* = − 40, *Y* = 30, *Z* = 17; these Talairach and Tournoux coordinates, and those reported in the text to follow, represent local maxima). This extended from the insula, through the inferior and middle frontal gyri into the ventral precentral gyrus. The second significant activation extended from the superior portion of the supramarginal gyrus (SMG) into the superior parietal lobule (SPL) and the precuneus (11.28cm^3^ volume; *X* = −29, *Y* = −67, *Z* = 40). Finally, significant activation was identified in the medial portion of the superior frontal gyrus (SFG), incorporating the anterior cingulate (7.37cm^3^ volume; *X* = 0, *Y* = 1, *Z* = 50). These data suggest that a left-lateralised network of three regions is engaged by deaf participants performing a phonological similarity judgement task, regardless of whether the task is performed in English or BSL.

#### Differences between the rhyme and location tasks and the effect of age of BSL acquisition in deaf participants

**Fig. 4:**
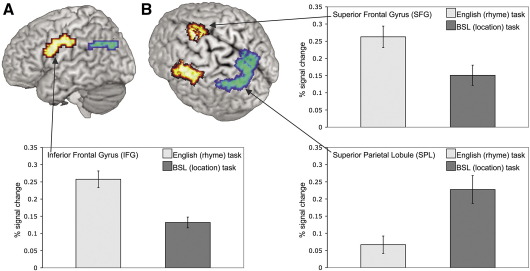
Main effect of task: rhyme versus location task in the deaf group only (*n* = 20; voxelwise *p* < 0.025; clusterwise *p* < 0.005). The left dorsal IFG and the medial portion of the SFG (shown in orange) were engaged to a greater extent during the rhyme than location task. The left SPL (shown in green/blue) was engaged to a greater extent during the location than rhyme task. View (A) provides the best illustration of the left IFG activation; view (B) provides the best view of the extent of activation in the SFG and the parietal lobe. Activated voxels up to 25mm beneath the cortical surface are displayed. Plots represent the mean % signal change across all voxels in the activated cluster across all participants. Error bars represent the standard error of the mean.

To determine the differences between the networks recruited during the rhyme and location judgement tasks and to examine the effect of age of signed language acquisition, a mixed-model ANOVA was conducted. This included Task (rhyme/location) as a within subjects factor and Age of BSL Acquisition (native/non-native) as a between subjects factor (voxelwise *p* = 0.025; clusterwise *p* = 0.005). The main effect of Task showed that two regions were recruited to a greater extent for the rhyme than location task. These were the left dorsal IFG, extending into the precentral gyrus (4.13cm^3^ volume; *X* = − 51, *Y* = − 4, *Z* = 40; BA 6), and the medial portion of the SFG, at the junction with the anterior cingulate (2.30cm^3^ volume; *X* = 0, *Y* = − 11, *Z* = 53; BA 6). In contrast, a region in the left parietal lobe was recruited to a greater extent for the location than rhyme task (see Fig. 4). This extended from the superior portion of the SMG, into the SPL and medially to include the precuneus (5.10cm^3^ volume; *X* = − 4, *Y* = − 70, *Z* = 43; BA 7).

**Fig. 5:**
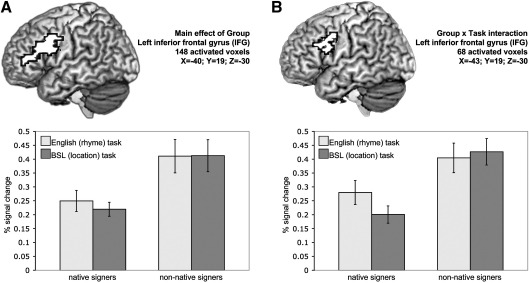
(A) Main effect of Group: non-native signers engaged the left inferior frontal gyrus to a greater extent than native signers. Critically this was the case during both the location and rhyme tasks (see text). (B) A significant Group × Task interaction was also identified in the left posterior IFG/precentral gyrus. Non-native signers recruited this region to a similar degree during both rhyme and location tasks. Native signers engaged this region more during the rhyme task, performed in English which was learned late, than the location task performed in their native language (see text). Activated voxels up to 20mm beneath the cortical surface are displayed. Plots represent the mean % signal change across all voxels in the activated cluster within each group of participants. Error bars represent the standard error of the mean.

No regions were recruited to a greater extent by native than non-native signers. In contrast, non-native signers recruited the left inferior frontal cortex to a greater extent than native signers (see Fig. 5A; 5.32cm^3^ volume; *X* = − 40, *Y* = 19, *Z* = 30). This activation extended from the IFG (BA 44/45), into the middle frontal gyrus and precentral gyrus. Follow-up analyses were conducted comparing native and non-native signers on the rhyme and location tasks separately (voxelwise *p* = 0.025; clusterwise *p* = 0.005). These analyses confirmed that non-native signers engaged the posterior IFG more than native signers, during *both* the location task (3.45cm^3^ volume; *X* = − 40, *Y* = 15, *Z* = 30) and the rhyme task (3.74cm^3^ volume; *X* = − 40, *Y* = 7, *Z* = 23).

A significant Group × Task interaction was also identified, the focus of which was at the junction of the left IFG (BA 44), the precentral gyrus (BA 6) and the middle frontal gyrus (BA 9; see Fig. 5B; 2.44cm^3^ volume; *X* = − 43, *Y* = 19, *Z* = 30). Follow-up analyses (voxelwise *p* = 0.025; clusterwise *p* = 0.005) demonstrated that there was no significant difference in the extent to which non-native signers recruited this region during the rhyme and location tasks. In contrast, native signers engaged this region more during the rhyme task, performed in English which they learned late, than the location task, performed in their native language (2.87cm^3^ volume; *X* = −47, *Y* = 0, *Z* = 36, precentral gyrus (BA 6)). The medial portion of the SFG, at the junction with the anterior cingulate, also demonstrated the same effect (2.16cm^3^ volume; *X* = 0, *Y* = 11, *Z* = 53).

#### Rhyme similarity judgements in hearing participants (voxelwise *p* = 0.025; clusterwise *p* = 0.01)

Hearing participants performing the rhyme task engaged four regions (see Table 3): [1] the left prefrontal cortex extending from the insula, through inferior and middle frontal gyri and into the precentral gyrus, [2] the superior portion of the SMG, extending into the SPL and medially into the precuneus, [3] the anterior medial portion of the SFG and superior portions of the anterior cingulate, and [4] the right inferior occipital gyrus extending into the fusiform gyrus and incorporating superior parts of the cerebellum.

The pattern observed in hearing participants performing the rhyme task was similar to that observed in deaf people performing the rhyme and location tasks. To clarify the overlap in regions recruited during phonological similarity judgements a conjunction analysis was conducted on the data from deaf and hearing participants performing the rhyme task and deaf participants performing the location task (voxelwise *p* = 0.05; clusterwise *p* = 0.01). Not surprisingly, given the individual group patterns, this analysis identified three regions as being significantly activated across all tasks/groups: the dorsal portion of the left IFG, extending into the middle frontal gyrus (14.81cm^3^ volume; *X* = −40, *Y* = 7, *Z* = 33 BA 44/9); the left SPL (5.82cm^3^ volume; *X* = −29, *Y* = −63, *Z* = 46 BA 7) and the medial portion of the SFG (5.61cm^3^ volume; *X* = 0, *Y* = 11, *Z* = 50 BA 6). These regions appear to make up a core network involved in phonological processing of both signed and spoken language, recruited by both deaf and hearing participants.

#### Deaf versus hearing participants performing rhyme task, matched for performance

To determine the effect of hearing status on the neural system supporting phonological processing, we contrasted activation patterns in deaf and hearing participants performing the rhyme task. Since group performance on the rhyme task was poorer in deaf than hearing participants, subsets of 12 participants were selected from each group who were matched on accuracy and reaction time on the rhyme task performed in the scanner. To further control for differences between deaf and hearing participants the subgroups were also matched for age, non-verbal IQ, reading age, and accuracy and reaction time on a more extensive test of rhyme awareness, run prior to the scan session. There were no significant differences between the two groups on any of these variables (all *p*-values > 0.1; see Table 4).

A between subjects ANOVA (voxelwise *p* = 0.05; clusterwise *p* = 0.005) indicated no regions in which hearing participants showed greater activation than deaf participants. The deaf subgroup showed greater activation than the hearing subgroup in the left IFG, extending into the middle frontal and the precentral gyri (7.01cm^3^ volume; *X* = −40, *Y* = 0, *Z* = 33; BA 44/6) and in a small portion of the SFG, at the junction with the anterior cingulate (1.29cm^3^ volume; *X* = − 4, *Y* = 4, *Z* = 50; BA 6/32). Further analyses confirmed that the same pattern was observed when deaf native signers and deaf non-native signers were compared separately to matched hearing participants. This suggests that combining native and non-native signers, in order to carefully match subgroups of deaf and hearing participants, did not influence the outcome of this analysis.

## Discussion

Our results demonstrate that a very similar neural network supports phonological similarity judgements made in both English and British Sign Language (BSL). Given that these languages operate in such different modalities, these data suggest that this phonological processing network is multimodal or possibly to some extent *supramodal*: that is, involving representations that in some way ‘transcend’ the sensory modalities (see Fowler, 2004 for discussion). This network, which was also recruited by hearing people making rhyme judgements, consists of the medial portion of the superior frontal gyrus (SFG), the left superior parietal lobule (SPL) incorporating the superior portion of the supramarginal gyrus (SMG), and, most extensively, the left posterior inferior frontal gyrus (IFG) extending into the ventral precentral gyrus. We do not argue that these regions are *dedicated* to phonological processing. Rather we argue that they act together as a network to support phonological similarity judgements and other linguistic and, it is likely, non-linguistic processes (see Corina and Knapp, 2006). Nevertheless, our data are consistent with prior demonstrations, concerning semantic and syntactic processing, that modality has relatively little influence on the neural systems that support language (Neville et al., 1998; Petitto et al., 2000; Braun et al., 2001; Emmorey et al., 2002; MacSweeney et al., 2002b; Corina et al., 2003; MacSweeney et al., 2006). Demonstrating this in the context of phonological processing is even more striking since awareness of phonology is more directly linked to sensory input (which differs for sign and speech) than either semantic or syntactic processing.

Although the observed network is recruited by both signed and spoken language, we demonstrate that it does not perform identically across languages or groups. Recruitment of different parts of this network is modulated by age of first language acquisition, language modality and hearing status. The impact of age of first language acquisition was explored by comparing deaf native and non-native signers. Non-native signers engaged the left posterior inferior frontal cortex to a greater extent than native signers. This was the case not only during the task performed in BSL, of which both groups had different language experience, but also during the task performed in English, of which both groups had similar experience. The differential recruitment of the left posterior IFG is even more striking given that native and non-native signers were matched on non-verbal IQ, English vocabulary score and reading age and that there were no significant group differences in accuracy or reaction time on the two phonological tasks. These are the first neuroimaging data to demonstrate the impact of age of acquisition of a first language in the brain. Lack of exposure to a fully accessible language early in life has implications for the neural systems supporting not only that language, but also for languages learned subsequently, whether signed or spoken. In conjunction with the behavioural data of Mayberry and colleagues (Mayberry et al., 2002; Mayberry and Lock, 2003) these data highlight the importance of early exposure to an accessible language for those born profoundly deaf. Even when signed, early language leads to the normal establishment of language systems that may then be used to facilitate a later learned language.

Enhanced recruitment of the left posterior IFG has previously been reported during grammaticality judgements performed by late in contrast to early learners of German (Wartenburger et al., 2003) and during semantic judgement tasks in low- in contrast to high-proficiency late language learners (Chee et al., 2001; Wartenburger et al., 2003). Regions within the left IFG are differentially modulated not only by age of language acquisition and proficiency level, but also by extent of language use (Perani et al., 2003), age at time of testing, and task demands (Tatsuno and Sakai, 2005). Thompson-Schill et al. (2005) argue that the left IFG is increasingly engaged as *selection* demands increase. In particular, it is argued that this region is involved in regulating the cognitive control necessary to resolve competition between incompatible responses (Snyder et al., 2007). It is possible that selection demands increase for bilinguals because responses from both first and second languages are available. This situation applies to native signers (first language: BSL; second language: English), but may apply to a greater extent to non-native signers. Deaf non-native signers have knowledge of both BSL and English; however, both languages are acquired late. Despite equivalent behavioural proficiency on our tasks, both languages are likely to be poorly established in non-native signers, leading to greater conflict between potential responses, possibly resulting in enhanced recruitment of the left IFG.

An alternative, phonology-specific argument can also be made for the role of the posterior IFG. It has been argued that different parts of the left IFG may show preferential engagement in different aspects of language processing: phonological (the dorsal region: BA 44/6), syntactic (the more anterior region: BA 45), and semantic (the ventral portion: BA 47) (Fiez, 1997; Price et al., 1997, Poldrack et al., 1999; Bookheimer, 2002; Devlin et al., 2003). While the baseline task used in the current study did not *require* picture name retrieval, given the relatively long presentation time (5s) it is likely that participants did name these stimuli (cf. Meyer and Damian, 2007). Furthermore, the network we identify in the current study has been reported in numerous previous studies of phonological processing involving written words (e.g., Lurito et al., 2000; Xu et al., 2001; Seghier et al., 2004). The most parsimonious interpretation of the current data is thus in terms of phonological processing. With regard to the left frontal cortex, as in previous studies, it was specifically the posterior portion of the IFG, extending into the ventral premotor cortex within precentral gyrus, that was the focus of activation involved in the phonological similarity matching tasks reported here. Moreover, this region was engaged to a greater extent by deaf participants during the rhyme than location task and more by deaf than hearing participants performing the rhyme task (see also Aparicio et al., 2007), even when behavioural performance was taken into account. To account for these findings, we suggest that when the auditory component of speech is absent, the articulatory/motoric component makes a greater contribution to speech-based phonological processes. A similar explanation may account for the observed increased involvement of the left IFG during reading in children with developmental dyslexia following phonological remediation (e.g., Temple et al., 2003). Further studies with deaf participants are underway to test this hypothesis. We also found that the posterior portion of the left IFG was engaged more by non-native than native signers during tasks performed in both languages. Broca's area, in left posterior IFG, is engaged in sign production, just as it is in speech production (Braun et al., 2001; Corina et al., 2003; Emmorey et al., 2007). One possibility that may account for the effect of age of first language acquisition in this region is that the articulatory component of both speech and sign is less established in deaf non-native than native signers, leading to enhanced recruitment of this region during *both* tasks.

From our data, it is not possible to distinguish between phonology-specific and cognitive control/selection demands accounts of the differential engagement of the left IFG by native and non-native signers. Indeed both accounts may apply since the area showing differential activation in all of the contrasts reported here involved both the posterior portion of the left IFG and the ventral premotor cortex, in the precentral gyrus. Snyder et al. (2007) propose that the left IFG is involved in cognitive control, while the ventral premotor cortex demonstrates phonology specific properties. The relative role of these regions in language processing, and phonological processing in particular, will be greatly informed by future studies examining different domains of language (phonology, syntax, semantics) within the same group of deaf late language learners, while manipulating age of acquisition and proficiency.

The left parietal lobe was also recruited during both the location task (deaf participants) and the rhyme task (deaf and hearing participants). In all groups and tasks this activation included the superior portion of the supramarginal gyrus (SMG), extending into the superior parietal lobule (SPL) and medially into the precuneus. Previous studies of rhyme judgement of written words by hearing adults also report activation of this area (Lurito et al., 2000; Xu et al., 2001; Seghier et al., 2004; Snyder et al., 2007). Nevertheless, the exact role of this region in phonological processing remains unclear. It has been proposed that this multimodal integration region may be recruited during mapping between orthographic and phonemic representations (Booth et al., 2002; Eden et al., 2004). The fact that the stimuli in the current study were pictures, not written words, does not necessarily pose a problem for this account (but see Katzir et al., 2005). Hearing adults have been shown to engage this region more than children during *auditory* rhyme decisions (Booth et al., 2004). In addition, deaf people are more accurate and faster to make rhyme judgements when the labels for picture stimuli share orthography, e.g., cat–mat, than when they do not, e.g., chair–bear (Sterne and Goswami, 2000). Both of these lines of evidence suggest that orthographic representations may be activated in both deaf and hearing participants when making spoken language phonological decisions in response to pictures. Further studies are needed to examine this hypothesis and the role of the left parietal lobe in phonological processing.

Whatever the functional role of the left parietal lobe in spoken language phonological processing, a growing number of studies suggest that this area may play a particularly important role in signed language processing (see Corina and Knapp, 2006). Perception of BSL sentences that involve spatial description engage the left inferior parietal lobule (IPL) and SPL to a greater extent than ‘non-spatial’ sentences in deaf native signers (MacSweeney et al., 2002a). Direct stimulation of the IPL induces phonological errors during sign production (Corina et al., 1999). Emmorey et al. (2007) have reported that sign production engages the left IPL (*X* = −60, *Y* = −25, *Z* = 27) and the left SPL (*X* = −26, *Y* = −51, *Z* = 54) more than speech production. Emmorey et al. (2007) propose that the left IPL may be involved in phonological processing while the left SPL may be involved in proprioceptive monitoring of motor output. In the current study, the observed greater activation in deaf signers during the location task than the rhyme task was located between the two foci reported by Emmorey et al. (2007) and incorporated the superior portion of the SMG in the IPL, extending into the SPL (*X* = −4, *Y* = −70, *Z* = 43). Given the nature of the current task, a phonological account of this activation seems more likely than an account based on proprioceptive monitoring. Corina and Knapp (2006) have argued that the left parietal lobe plays a greater role in signed than spoken language processing because signed language can build on the “...prior existence of a general human system for manual action observation and production” (p. 537). Portions of the parietal lobe are particularly attuned to the location of the hands/body in space (Gerardin et al., 2000; Hermsdorfer et al., 2001). Accordingly, this region may be particularly engaged in tasks that focus on these spatial relationships, including those involving sign phonology. Whether this activation is specifically related to linguistic processing or to more general processing of body-related information requires further investigation, using linguistic tasks exploring both comprehension and production, and non-linguistic tasks.

Examining other parameters of sign phonology, e.g., movement and handshape, is also required. It is not possible to claim a straightforward parallel between rhyme in spoken language and any of the parameters of sign phonology. It has been suggested that when any sign parameter is shared, this is more analogous to alliteration than to rhyme (Sutton-Spence, 2004). Location was chosen in the current study because it has been demonstrated to be important in judging sign similarity (Hildebrandt and Corina, 2002) and because, among the phonological parameters of signed languages, it appears to have the smallest inventory, as do vowels, which are the nucleus of the syllable and crucial for rhyme in spoken languages. However, sign phonologists may argue that movement is more analogous to vowels with respect to syllable structure because a sign is ill-formed without movement, just as a syllable is ill-formed without a vowel (Brentari, 1999; Sandler and Lillo-Martin, 2006). Whether different patterns of activation are observed when different sign parameters are examined or whether any form of sublexical analysis of signs elicits activation in the network reported here remains to be seen.

In summary, these data suggest that phonological processing, at least in the context of a phonological similarity judgement task, is to some degree *supramodal.* We show that a similar neural network supports phonological processing of both signed and spoken language in signed language users born profoundly deaf. Furthermore, this network was also engaged by hearing non-signers performing the similarity judgement task in English. However, different parts of this network were differentially weighted depending upon language modality, hearing status and, most importantly, age of first language acquisition. These data highlight the importance of learning a language, whether signed or spoken, early in life. Early acquisition of a first language is critical not only for processing that language, but also appears to form a base on which subsequently learned languages can successfully build.

## Figures and Tables

**Table 1:** Participant characteristics: mean [S.D.] and range of age, non-verbal IQ (percentile), speechreading, reading age, and English productive vocabulary

	Age	NVIQ (percentile)	Test of Adult Speechreading (max = 40)	Reading age	Vocabulary (max = 30)
All deaf signers (*n* = 20; male = 8)	34;08 [9;06] 22;01–54;08	85.8 [14.4] 50–99	31.7 [4.4] 22–39	15;06 [3:04] 8;04–20;00	26.0 [2.96] 19–30
*Deaf native signers (*n* = 12; male = 5)*	*31; 05 [9;05] 22;01–54;08*	*85.0 [16.4] 50–99*	*31.3 [3.2] 25–36*	*15;07 [1;11] 11;09–18;04*	*26.4 [2.15] 22–30*
*Deaf non-native signers (*n* = 8; male = 3)*	*39; 06 [7;08] 30;04–49;08*	*87.0 [11.7] 63–95*	*32.3 [5.9] 22–39*	*15;04 [5;00] 8;04–20;00*	*25.4 [4.0] 19–29*
Hearing non-signers (*n* = 24; male = 13)	35;03 [8;10] 22;01–55;04	82.6 [19] 25–100	24.1^⁎^ [4.5] 18–33	18;10 [2;03] 15;00–23;00	28.8 [1.46] 24–30

^⁎^Only 16 of the 24 hearing participants completed the Test of Adult Speechreading.

**Table 2:** Behavioural data: mean [S.D.] accuracy (Acc.; max = 30) and reaction times (RT; seconds) on rhyme, location and ‘same picture?’ tasks by each group

	Rhyme?		Location?		Same picture?	
	Acc.	RT	Acc.	RT	Acc.	RT
All deaf signers (*n* = 20)	26.2 [2.20]	2.5 [0.41]	25.0 [3.21]	2.8 [0.42]	29.5 [0.55]	1.2 [0.25]
*Deaf native signers (*n* = 12)*	*26.6 [2.00]*	*2.5 [0.40]*	*25.8 [2.21]*	*2.8 [0.43]*	*29.5 [0.58]*	*1.1 [0.24]*
*Deaf non-native signers (*n* = 8)*	*25.5 [2.40]*	*2.6 [0.46]*	*23.7 [4.20]*	*2.9 [0.42]*	*29.6 [0.52]*	*1.2 [0.28]*
Hearing non-signers (*n* = 24)	28.3 [1.29]	2.4 [0.41]	–	–	29.4 [0.97]	1.2 [0.31]

**Table 3:** Activation, relative to the ‘same picture?’ control task, during the (A) location task in the deaf group; (B) rhyme task in the deaf group; (C) rhyme task in the hearing group

	Volume (cm^3^)	*X*	*Y*	*Z*	BA
(A) Location task > baseline (deaf group; *n* = 20)					
Left IFG	16.50	− 43	15	30	9/44
Left SPL/precuneus	9.67	− 29	− 63	36	7
Medial SFG/anterior cingulate	4.10	4	11	46	6/32
(B) Rhyme task > baseline (deaf group; *n* = 20)					
Left IFG	15.06	− 40	4	33	6/44
Left SPL/precuneus	6.11	− 25	− 67	40	7
Medial SFG/anterior cingulate	7.04	0	7	50	6/32
(C) Rhyme task > baseline (hearing group; *n* = 24)					
Left IFG	15.38	− 40	7	26	44
Left SPL/precuneus	6.00	− 25	− 59	40	7
Medial SFG/anterior cingulate	5.39	0	19	46	6/32
Right inferior occipital gyrus	2.87	40	− 74	− 7	19

Coordinates (Talairach and Tournoux, 1988) report maxima of 3D clusters. Voxelwise *p* < 0.025; clusterwise *p* < 0.01.

**Table 4:** Matched subgroups: Participant characteristics and accuracy and reaction time (RT) on the rhyme task performed in the scanner and on a larger assessment of rhyme awareness (same procedure) performed out of the scanner, in 12 deaf and 12 hearing participants

	Deaf (*n* = 12)	Hearing (*n* = 12)
Age	34;05 [10;02] 22;01–54;08	33;06 [8;00] 22;01–48;06
Reading age	17;03 [1;08] 15;00–20;00	17;01 [1;05] 15;00–19;00
NVIQ	88th percentile [12.3] 63–100	76th percentile [21.9] 25–99
Vocabulary (max = 30)	27.3 [1.8] 24–30	28.2 [1.8] 24–30
Rhyme accuracy in scanner	90% [3.3] 87–97%	93% [4.0] 87–97%
Rhyme RT in scanner	2.5 s [0.3] 1.9–3.1	2.4 s [0.5] 1.5–3.0
Rhyme accuracy out of scanner	90% [4.5] 82–94%	93% [5.0] 85–100%
Rhyme RT out of scanner	3.1 s [0.6] 2.1–4.0	2.7 s [0.8] 1.7–4.3

Mean [S.D.] and range are reported. There were no significant differences between the two groups on any of the variables [*p* > 0.1].
